# Comparative Photo‐Induced Aging of Poly(Butylene Adipate‐co‐Terephthalate) and Polystyrene Microplastics and their Divergent Affinities for Tetracycline in Aquatic Environments

**DOI:** 10.1002/open.202500243

**Published:** 2025-06-25

**Authors:** Sadam Hussain Tumrani, Zeenat Naz, Razium Ali Soomro, Mohamed E. Khalifa, Gaber A. M. Mersal, Ahmed M. Fallatah, Selcan Karakuş

**Affiliations:** ^1^ Key Laboratory of Water Environment Simulation School of Environment Beijing Normal University Beijing 100875 P. R. China; ^2^ The Key Laboratory of Water and Sediment Sciences Ministry of Education School of Environment Beijing Normal University Beijing 100875 P. R. China; ^3^ Dept. of Mechanical and Aerospace Engineering Sapienza University of Rome Via Eudossiana 18 00184 Rome Italy; ^4^ State Key Laboratory of Organic‐Inorganic Composites Beijing Key Laboratory of Electrochemical Process and Technology for Materials Beijing University of Chemical Technology Beijing 100029 China; ^5^ Department of Chemistry College of Science Taif University P.O. Box 11099 Taif 21944 Saudi Arabia; ^6^ Department of Chemistry Faculty of Engineering Istanbul University‐Cerrahpaşa 34320 Istanbul Turkey; ^7^ Health Biotechnology Joint Research and Application Center of Excellence Esenler 34220 Istanbul Turkey

**Keywords:** emerging pollutants, microplastics, surface water, tetracycline, UV aging

## Abstract

Microplastics (MPs) experience photo‐induced surface modification in sunlit waters, yet the implications for contaminant binding differ fundamentally for biodegradable and conventional MPs. To simulate submerged aging, biodegradable poly (butylene adipate‐co‐terephthalate) (PBAT) and nondegradable polystyrene (PS) are exposed to ultraviolet A irradiation and river water for 30 days. Aged PBAT shows significant surface oxidation, including a 16% decrease in carbonyl index, a reversal of ζ‐potential from slightly positive to −50 mV, and an increase in tetracycline (TC) sorption kinetics. In contrast, the nondegradable analog (PS) displays only modest oxidation (ΔCI ≈ 6%), a smaller charge shift, and a lower capacity increase (*q*_m_ ≈ 33 mg g^−1^). Maximum TC uptake occurs at pH ≈ 7, whereas modest salinity increments (0.010–0.0105 M) attenuate retention, confirming the controlling influence of electrostatic forces. Fluorescence quenching (86% vs 74% for PBAT and PS) and Akaike information criterion/Bayesian information criterion‐ranked model fits indicate that hydrogen bonding and electrostatic attraction dominate on the biodegradable surface, whereas π–π and hydrophobic interactions on the PS. This work confirms that submerged photo‐oxidation turns biodegradable MPs into reactive, salinity‐sensitive antibiotic sinks while conventional MPs remain inert transport vectors, emphasizing the need for MP‐specific risk assessment and antibiotic pollution mitigation in aquatic settings.

## Graphical abstract

UV‐water aging endows biodegradable poly (butylene adipate‐co‐terephthalate) (PBAT), unlike polystyrene, with abundant polar groups that markedly enhance its interaction with tetracycline and subsequent sorption, highlighting distinct antibiotic retention and release behavior for biodegradable versus conventional microplastics.

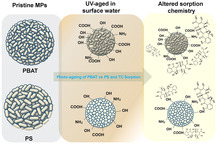

## 1 Introduction

Microplastics (MPs), defined as plastic particles smaller than 5 mm, have emerged as a significant environmental concern due to their pervasive presence in terrestrial, freshwater, and marine ecosystems.^[^ ^1^, ^2^, ^3^ ^]^ Their persistence and ability to adsorb and transport contaminants raise serious concerns about their potential ecological and human health risks.^[^ ^4^ ^]^ MPs originate from industrial discharges, urban runoff, and agricultural activities, continuously entering natural waters where their diminutive size, large surface area, and hydrophobic nature facilitate interactions with pollutants.^[^ ^5^, ^6^, ^7^, ^8^ ^]^ These interactions enhance pollutant bioavailability, disrupt microbial activity and nutrient cycling, and heighten risks of bioaccumulation in aquatic food chains.^[^ ^9^, ^10^, ^11^ ^]^

Polystyrene (PS) and poly (butylene adipate‐co‐terephthalate) (PBAT) represent two contrasting polymer types found in aquatic environments. PS is a lightweight, rigid thermoplastic used in food packaging and single‐use items, known for its durability against degradation.^[^ ^12^ ^]^ In contrast, PBAT is a copolyester designed for rapid hydrolytic and enzymatic breakdown, commonly found in compostable films and bags.^[^ ^13^ ^]^

Recent evidence suggests that the ecological risks associated with PS and biodegradable PBAT MPs are complementary rather than contradictory. Lu et al.^[^ ^14^ ^]^ found that PS‐MPs rapidly accumulate in the gut, liver, and gonads of zebrafish, particularly under nutrient‐rich conditions (such as a high‐fat diet), leading to altered lipid metabolism and systemic retention. At the same time, Xie et al.^[^ ^15^ ^]^ demonstrated that PBAT‐MPs result in comparable levels of tissue accumulation in embryonic and juvenile zebrafish, despite the polymer's nominal biodegradability. From a toxicological perspective, exposure to PS enhances the toxicity of co‐contaminants; for example, it facilitates the transfer of microcystin‐LR (stands for leucine (L) and arginine (R)) into reproductive tissues and exacerbates endocrine disruption.^[^ ^16^ ^]^ Meanwhile, PBAT fragments induce oxidative stress, neurobehavioral deficits, and delayed hatching, effects that are comparable to, or even exceed, those caused by PS when administered at environmentally relevant concentrations.^[^ ^17^ ^]^

Photochemical weathering is recognized as the principal environmental driver of surface transformation for both recalcitrant and biodegradable polymers.^[^ ^18^ ^]^ Lambert and Wagner^[^ ^19^ ^]^ first demonstrated that UV‐B exposure increases the carbonyl index of PS by >300% within 30 d, generating nano‐scale cracks that treble its sorption capacity for fluoroquinolones. Subsequent work by Kijchavengkul, T. et al.^[^ ^20^ ^]^ showed that PBAT loses up to 25% of its mass after 21 d of simulated solar irradiation, leaving highly oxidized fragments enriched in carboxyl and vinyl‐ketone groups. Zheng, M. et al.^[^ ^21^ ^]^ extended these observations to antibiotics, reporting a subsequent rise in ciprofloxacin uptake on UV‐aged polylactic acid relative to pristine polyethylene, while Zhang, X. et al.^[^ ^22^ ^]^ confirmed that photo‐oxidized PS becomes a persistent hot spot for tetracycline sorption and trophic transfer.

Aging studies reveal that weathered PBAT has a higher antibiotic adsorption capacity than conventional polyethylene or pristine PS due to surface cracking and increased crystallinity.^[^ ^23^ ^]^ While photo‐oxidized PS remains a durable site for contaminant sorption, both surfaces promote dense biofilms that accelerate the transfer of antibiotic‐resistance genes (ARGs), albeit through different mechanisms. Yang et al.^[^ ^24^ ^]^ tied hydrophobic PS to increased ARGs in marine biofilms, while Zheng et al.^[^ ^25^ ^]^ linked biodegradable MPs like PBAT to high‐risk ARGs and pathogens in freshwater, likely due to their rough surfaces and leachates. Recent research suggests that polymer diversity, rather than degradability, is more closely related to ARG and virulence factor abundance.^[^ ^26^ ^]^ These findings challenge the idea that biodegradability reduces ecological risk, as PBAT's faster fragmentation can enhance sorption sites and microbial interaction, potentially matching or exceeding PS in promoting bioaccumulation and ARG dissemination.

Antibiotics, such as tetracycline (TC), represent a significant class of emerging contaminants due to their widespread use in human and veterinary medicine, persistence in wastewater systems, and detection in aquatic environments at concentrations up to 1322 ng L^−1^.^[^ ^27^, ^28^ ^]^ This prevalence is largely attributed to their widespread use in both human and veterinary medicine and their persistence within wastewater systems.^[^ ^29^, ^30^ ^]^ Antibiotics like sulfamethoxazole, ciprofloxacin, tetracycline, and amoxicillin in water systems reflect their extensive use in infection treatment.^[^ ^31^, ^32^ ^]^ For example, sulfonamides have been found at concentrations up to 13,800 ng L^−1^ in Kenya and 3508 ng L^−1^ in Vietnam.^[^ ^33^ ^]^ Among these antibiotics, TC is one of the most commonly detected, with concentrations reaching up to 1322 ng L^−1^ in surface waters and a 100% detection frequency in certain regions, such as the Yangtze River Delta.^[^ ^34^ ^]^

These antibiotics can readily encounter suspended MPs, where photo‐oxidation‐induced surface groups may act as sorption sites that modulate antibiotic transport, persistence, and bioavailability.^[^ ^35^ ^]^ Although significant research is dedicated to exploring the dynamics of antibiotics with MPs to elucidate the actual behavior of MPs toward contaminants, most work decouples microplastic weathering from the aqueous matrices in which it actually occurs, examining either dry UV‐irradiation or aging in surface water.^[^ ^36^ ^]^ Consequently, the interplay between submerged photoaging in nutrient‐ and ion‐rich wastewater and subsequent antibiotic sorption remains poorly resolved, particularly for biodegradable polymers whose oxidation proceeds more rapidly than that of conventional plastics.

Herein, contrasting environmental roles of biodegradable and conventional MPs (PBAT and PS) in antibiotic‐rich environments are investigated through a systematic exposure of MPs under simulated aquatic conditions, a setting that couples photochemical attack with the shielding, ionic, and organic situation typical of surface waters. A 30‐day aging protocol combining UV irradiation during continuous immersion in surface‐derived wastewater confirmed distinct surface oxidation characteristics of aged MPs (16% carbonyl index reduction, 55 mV ζ‐potential shift), while conventional MPs exhibit minimal structural alteration. These contrasting surface transformations govern tetracycline (TC) interaction. The pH‐ and salt‐controlled adsorption‐desorption tests reveal that hydrogen bonding and electrostatic attraction dominate on oxidized PBAT, doubling its capacity (*q*_m_ ≈ 100 mg g^−1^) and tripling uptake rates, while π–π/hydrophobic forces remain primary on PS. Model selection by Akaike information criterion/Bayesian information criterion (AIC/BIC) further confirms the mechanistic split where under alkaline conditions, PBAT releases ≈78% of sorbed TC versus ≈62% from PS, indicating that biodegradable MPs act as short‐lived hotspots, whereas conventional MPs function as persistent transport vectors, evidence that polymer‐specific risk assessments are essential for antibiotic‐rich waters.

## 2 Results and Discussion

### 2.1 Physicochemical Changes in PBAT and PS after Photoaging in Surface Water

**Figure 1:**
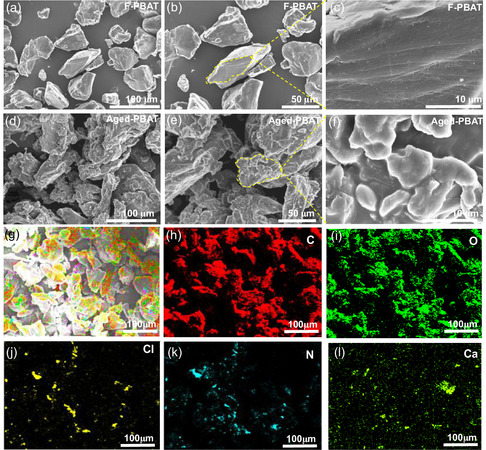
SEM images illustrating the morphological changes in a–c) fresh and d–f) aged PBAT and corresponding EDS mapping of g–l) aged PBAT highlight the variations in elemental distribution composition.

**Figure 2:**
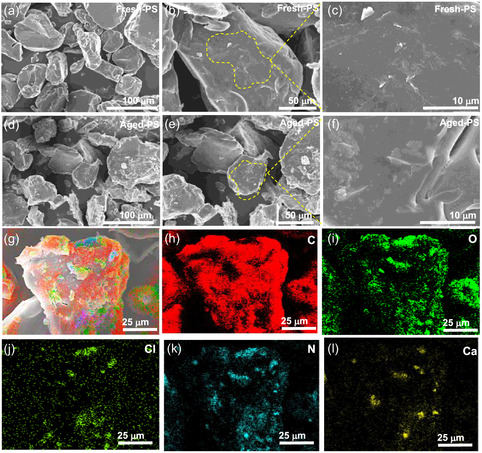
SEM images showing morphological changes in a–c) fresh and d–f) aged PS with corresponding EDS mapping of g–l) aged PS showing variations in elemental composition resulting from the aging process.

Biodegradable and nondegradable MPs were aged in surface water for 30 days under UV exposure to achieve sufficient aging to evaluate their interactional behavior with TC antibiotic.^[^ ^22^ ^]^ The water quality parameters and metal ion concentrations are provided in Table S1 and S2, Supporting Information. The metal ions can enhance the hydrolysis of MPs, whereas UV irradiation accelerates this process by generating free radicals and promoting polymer chain scission ester.^[^ ^37^, ^38^ ^]^ Comparative scanning electron microscope (SEM) analysis of fresh and aged MPs showed distinct changes post 30 days of age where, unlike fresh PBAT, which exhibits smooth and intact surfaces (**Figure** **1**a–c), aged PBAT, exhibits substantial surface roughness, cracking, and fragmentation (Figure 1d–f). This indicates a substantial effect of UV radiation and metal‐ion catalysis on PBAT morphology, which is anticipated to increase surface area and potential functionalization resulting in promoted interaction with antibiotics.^[^ ^38^ ^]^ Compared to PBAT, aged PS exhibits minimal changes in surface morphology (**Figure** **2**a–f), suggesting that submerged conditions and UV exposure had a relatively weaker impact on its structural integrity, where the smoother, less altered surface of aged PS indicates its greater photo‐oxidation resistance, consistent with its known stability under UV conditions.^[^ ^39^ ^]^ The energy‐dispersive spectroscopy (EDS) analysis further validated the involvement of metal ions in facilitating hydrolysis under UV irradiation. In contrast to fresh MPs (Figure S1, Supporting Information), which prominently exhibit carbon and oxygen as the primary elements in both PBAT and PS, the aged counterparts (Figure 1g–l and 2g–l) show additional surface‐bound elements, such as calcium (Ca), chlorine (Cl), and nitrogen (N). The presence of these metal ions is associated with adsorption from surface water, highlighting their potential influence on the oxidation‐induced functionalization of the polymer surface.^[^ ^40^ ^]^ The presence of the N element further indicates the initial stages of microbial colonization, indicating an increase in the bioavailability of the aged MPs.^[^ ^41^, ^42^, ^43^ ^]^ The differences in degradation are corroborated by the MP particle size distribution (Figure S2, Supporting Information), where aged PBAT exhibits a greater reduction and larger distribution of particle size compared to PS owing to its biodegradable nature, which enables relatively stronger bond scission and surface oxidation under aging conditions.^[^ ^44^ ^]^

**Figure 3:**
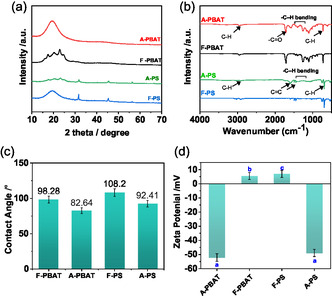
a) XRD patterns that compare the crystallinity of fresh and aged PBAT and PS, b) FTIR spectra compare the difference of surface functionalization between fresh and aged PBAT and PS, and corresponding c) ζ‐potential of fresh (F‐PBAT, F‐PS) and UV‐aged (aged‐PBAT, aged‐PS) MPs (pH 7, 25 °C). Bars represent mean ± SD (*n* = 3) and letters above bars denote statistical grouping: columns that share a letter do not differ significantly (one‐way ANOVA followed by Tukey's HSD, *p* ≥ 0.05), d) contact angles changes depicting the change in surface wettability between fresh and aged PBAT and PS with error bars representing mean ± SD (*n* = 3).

X‐ray diffraction (XRD) analysis further confirms this distinct degradation behavior, with aged PBAT exhibiting diffraction peaks that are relatively broad and less intense compared to PS, indicating the former's reduced crystallinity (**Figure** **3**a).^[^ ^45^ ^]^ The reduced crystallinity in the case of aged PBAT compared to PS is attributed to relatively stronger chain scission and oxidation, likely accelerated by hydroxyl radicals (·OH) produced via photo‐Fenton reactions under submerged conditions.^[^ ^46^, ^47^ ^]^

Fourier‐transform infrared (FTIR) analysis (Figure 3b) further assessed the change in functional groups over aged PBAT and PS during 30 days of exposure to UV irradiation in surface wastewater. Fresh PBAT exhibits characteristic ester‐related peaks, notably the carbonyl (C=O) stretch near 1725 cm^−1^ and C—H stretching (2850–2950 cm^−1^) from alkyl chains, typical of its hydrophobic nature.^[^ ^48^ ^]^ Fresh PS, in contrast, shows similar C—H stretches but lacks prominent C=O peaks, reflecting its nondegradable and more stable structure.^[^ ^16^ ^]^ Upon aging, MPs show marked changes in their functional group configuration. In the case of aged PBAT, the ester peaks at 1725 cm^−1^ decrease significantly, while new peaks appear around 1700–1800 cm^−1^, indicating the formation of carbonyl groups due to photo‐oxidation.^[^ ^49^ ^]^ Although PS shows a similar profile, less pronounced changes are evident with a slight shift in its C=O stretching band, suggesting surface oxidation, though it remains more stable than PBAT.^[^ ^50^ ^]^ The increased —OH stretching vibrations in the case of aged MPs indicate enhanced hydrophilicity, particularly for PBAT compared to PS. These newly generated —COOH/—OH groups on PBAT deprotonate above pH ≈ 5, imparting a higher negative surface charge that favors electrostatic attraction and hydrogen bonding with the zwitterionic or anionic forms of TC, whereas the largely hydrophobic PS surface relies mainly on weaker π–π interactions. This is also corroborated by contact angle measurements where PBAT falls from 98.3° ± 1.23 to 82.6° ± 1.17 while PS declines from 108.2° ± 1.67° to 92.4° ± 1.35 (Figure 3c and S3, Supporting Information). This change in wettability is consistent with FTIR where stronger loss of ester peaks (1725 cm^−1^) and a more pronounced emergence of carbonyl/–OH bands (1700–1800 and 3200–3400 cm^−1^) in PBAT confirms higher density of polar groups, suitable for enhancing the interaction with TC.^[^ ^51^ ^]^ Moreover, the carbonyl index (CI), further confirms a higher degree of degradation for aged PBAT, with CI changing from 0.7 ± 0.05 (fresh) to 0.59 ± 0.04 (aged) compared to PS (0.65 ± 0.02 to 0.61 ± 0.04) (Figure S4, Supporting Information), confirming substantial oxidative degradation of PBAT compared to PS, and thus, stronger surface functionalization.^[^ ^49^ ^]^ The change in surface characteristics is further corroborated by zeta potential measurements, where PBAT changes from +5.21 ± 1.23 to −50.28 ± 1.51 mV, a 55 mV shift, whereas PS moves from +8.64 ± 2.32 to −48.48 ± 1.22 mV, a 57 mV shift (Figure 3d). Although the magnitude is similar, PBAT crosses the zero‐charge point earlier and exhibits the larger contact‐angle drop, signifying a denser accumulation of carbonyl and —OH groups (evidenced by new FTIR bands (Figure 3b). Consequently, aged PBAT becomes the more hydrophilic, functionally rich surface, better suited for electrostatic and hydrogen‐bond interactions with TC than aged PS, which remains comparatively less polar.^[^ ^40^ ^]^

**Figure 4:**
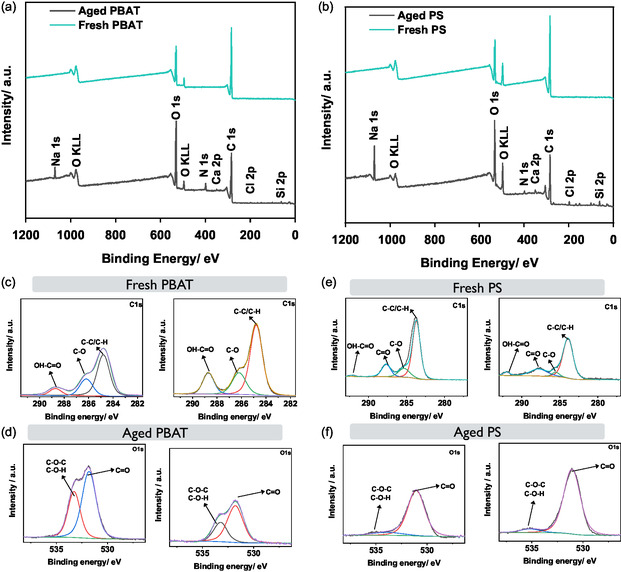
a,b) Survey spectra illustrating the fluctuation in the O/C ratio for both fresh and aged PBAT and PS, c,d) the deconvoluted C 1s and O 1s spectra for fresh and aged PBAT, and e,f) fresh and aged PS illustrating the alterations in functional group density due to the aging process.

X‐ray photoelectron spectroscopy (XPS) is conducted for both PBAT and PS in their fresh and aged states to further validate the change in surface functional groups of MPs. XPS of fresh PBAT and PS exhibits dominant C 1s peaks corresponding to C—C/C—H bonds, reflecting their hydrocarbon‐based structures (**Figure** **4**a,b). Fresh PBAT shows a marginally higher oxygen contribution than PS due to its ester (—COO—) linkages, while fresh PS primarily consists of aromatic carbon (C—C) with limited oxygen functionalities.^[^ ^44^ ^]^ After UV aging in surface water, both MPs show new peaks related to environmental elements such as Cl, Ca, and N, confirming interactions with dissolved ions.^[^ ^52^ ^]^

High‐resolution C 1s spectra of PBAT (Figure 4c,d) demonstrate a notable shift after aging, with an increased presence of C=O (carbonyl) and O—C=O (carboxylate) species, which is indicative of oxidative degradation and hydrolytic scission.^[^ ^52^ ^]^ These oxygenated functional groups increase the hydrophilicity of PBAT, improving its capacity for pollutant binding via hydrogen bonding and electrostatic interactions. In contrast, aged PS shows a minor increase in C—O and C=O peaks, indicating that oxidation is limited and does not significantly disrupt the polymer backbone (Figure 4e,f). This suggests that, while PBAT undergoes extensive structural changes, PS remains largely resistant to bulk oxidative degradation, with only surface‐level oxidation occurring. The O 1s spectra further corroborate these findings, showing that aged PBAT has significantly increased C=O and O=C—O contributions, aligning with ester hydrolysis and oxidative fragmentation (Figure 4d).^[^ ^53^, ^54^ ^]^ In contrast, PS exhibits minimal variations in the O 1s profile, with a slight rise in surface oxidation but no substantial modifications to the polymer backbone. Despite the observed decrease in the CI with aging, the escalating oxygen‐to‐carbon (O/C) atom ratio from 0.68 ± 0.02 (fresh PBAT) to 1.09 (aged PBAT) and from 0.98 ± 0.04 (fresh PS) to 1.06 (aged PS) validates the presence of abundant oxygen‐related functional groups on aged MPs.^[^ ^55^, ^56^ ^]^ This increase in the O/C ratio further reinforces the increased polarity and surface reactivity of both PBAT and PS after aging.^[^ ^52^ ^]^ However, PBAT exhibits a more pronounced transformation due to the combination of photo‐oxidative degradation and hydrolytic scission than its PS counterpart.^[^ ^51^ ^]^

### 2.2 Kinetic and Isotherm Behavior of Aged MPs toward TC

**Figure 5:**
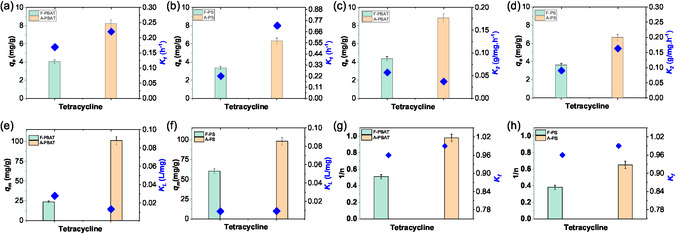
Bar graphs illustrating a,b) pseudo‐first (PFO) and c,d) second‐order (PSO) kinetics of TC over fresh and aged PBAT and PS and corresponding e,f) Langmuir and g,h) Freundlich model fittings.

The adsorption dynamics between TC and aged MPs offer key insights into their interactions.^[^ ^24^ ^]^ Kinetic and isotherm modeling confirmed distinct behavior of aged MPs toward TC. Evidently, aging in wastewater under UV‐irritation enhanced both the kinetics and adsorption capacity of MPs, with PBAT exhibiting improved TC adsorption kinetics compared to PS (**Figure** **5**a,b and Table S3, Supporting Information). For aged PBAT, the pseudo‐second‐order (PSO) model showed a strong fit (*R* ^2^ = 0.95–0.98), suggesting chemisorption as a dominant mechanism, driven by enhanced chemical interactions with adsorption capacity increased from 4.36 mg g^−1^ (fresh) to 8.84 mg g^−1^ (aged) (Table S3, Supporting Information). However, model evaluation using AIC and BIC favors PSO based on the residual sum of squares (RSS) values. The RSS for pseudo‐first order (PFO; 5.49 for fresh PBAT) is significantly lower than for PSO (63.84), with corresponding AIC (−0.43) and BIC (−0.04) values also lower than PSO (AIC = 21.63, BIC = 22.02) (Table S7, Supporting Information). This indicates that, despite PSO's better *R* ^2^ value, the PFO model more accurately captures interaction behavior over time. In addition, aged PS showed a modest increase in adsorption kinetics, with its PFO rate constant (*k*_1_) rising from 0.22 h^−1^ (fresh) to 0.72 h^−1^, and adsorption capacity increasing from 3.36 mg g^−1^ (fresh) to 6.32 mg g^−1^ (aged) (Figure 5b and Table S3, Supporting Information). Although the PSO model fits well (*R* ^2^ ≈ 0.99), interactions for PS remain weaker, driven by hydrophobic forces and electrostatic interactions. The model comparison further confirms PFO's superiority with a lower RSS (3.67) versus PSO (48.65), and favorable AIC (−4.058) and BIC (−3.66) values (Table S7, Supporting Information). The fitting profiles of PFO and PSO, along with their reference observed and predicted adsorption values, are provided in Figure S5 and Table S5–6, Supporting Information. Based on AIC and BIC values, PFO models better describe the kinetics of both aged PBAT and PS when considering the full adsorption time course where the primary adsorption mechanism, although governed by external‐film mass transfer, chemisorption becomes relatively dominant at later stages of adsorption.

The adsorption capacity of TC over aged MPs, particularly PBAT follows the Langmuir model with a substantial increase in monolayer adsorption capacity (*q*_m_) from 23.59 mg g^−1^ (fresh) to 101 mg g^−1^ (aged), indicating that aging in surface water under irradiation significantly enhances the number of available binding sites (Figure 5e,f and Table S4, Supporting Information). This increase aligns with abundant surface functionalization (Figure 3b), allowing for more efficient adsorption of TC through stronger chemical interactions, as supported by the PFO kinetic results. The Langmuir constant (*K*_L_) for aged PBAT decreased from 0.0278 to 0.0133 L mg^−1^, reflecting a reduction in per‐site strength but increased overall capacity, a trend consistent with greater surface reactivity following UV aging. However, PS adsorption behavior follows the Freundlich model, suggesting multilayer adsorption, driven by weaker, nonspecific surface interactions (Figure 5e–g). Aged PS showed a moderate increase in *q*_m_ from 60.34 to 98 mg g^−1^, demonstrating that surface oxidation enhances adsorption capacity, albeit to a lesser degree than PBAT (Table S4, Supporting Information). The *K*_L_ for PS remained nearly constant (0.0091–0.0095 L mg^−1^), indicating that aging did not significantly alter the adsorption mechanism, which remains dominated by hydrophobic and π–π interactions (Figure 5f). This is consistent with the kinetic analysis, where aged PS showed only modest increases in rate constants (*k*_1_) compared to PBAT (Table S3, Supporting Information). Additionally, Freundlich constant (*K*_f_) values for PS increased from 0.38 to 0.65 ((mg g^−1^) (L mg^−1^)1/*n*)), reflecting weaker adsorption interactions compared to PBAT (*K*_f_ rises from 0.51 to 0.90), which further supports the dominance of weaker interactions on PS surfaces (Figure 5h and Table S4, Supporting Information). Moreover, the (1/*n*) for both PBAT and PS approached 1, indicating favorable adsorption mechanisms. Overall, aged PBAT experiences more significant chemical transformation through aging, leading to stronger interaction with TC, while aged PS maintains weaker surface interactions even after oxidation. The contrasting behaviors highlight the importance of surface configuration in pollutant binding, revealing that aged PBAT exhibits enhanced sequestration efficiency but risks secondary contamination through fragmentation. In contrast, PS maintains structural stability, acting as a persistent pollutant vector rather than an active sorbent.

### 2.3 Interaction Mechanism of Tetracycline with Aged MPs

The mechanism of interaction of TC with aged PBAT and PS can reveal significant information regarding the long‐term behavior of MPs within the antibiotic‐rich environment.^[^ ^21^ ^]^ XRD analysis (Figure 3b) and CI (Figure S4, Supporting Information) support decreased crystallinity and increased functional groups in both PBAT and PS upon aging, which is favorable for TC adsorption. Post TC‐adsorbed FTIR analysis of aged MPs (Figure S6, Supporting Information) confirms a shift in the C=O stretching band from 1725 to 1600 cm^−1^, particularly for PBAT indicative of hydrogen bonding and electrostatic interactions with TC.^[^ ^23^ ^]^ The broad N–H stretching peak further supports hydrogen bonding, consistent with PSO kinetics, suggesting chemisorption as the dominant mechanism.^[^ ^57^ ^]^ In contrast, aged PS showed no significant shift in the C=O stretch, indicating weaker adsorption driven by hydrophobic interactions and physisorption, as corroborated by the PFO model (Table S5, Supporting Information). Although aged PS shows a more modest increase in adsorption capacity (from 3.36 to 6.32 mg g^−1^) (Table S4, Supporting Information), confirming that oxidation improves reactivity, surface interactions remain less robust than PBAT. The variation in interaction strength is further supported by UV–vis spectral changes, where aged PBAT shows a greater reduction in peak intensity compared to aged PS, indicating a stronger adsorption capacity for PBAT (Figure S7, Supporting Information).

**Figure 6:**
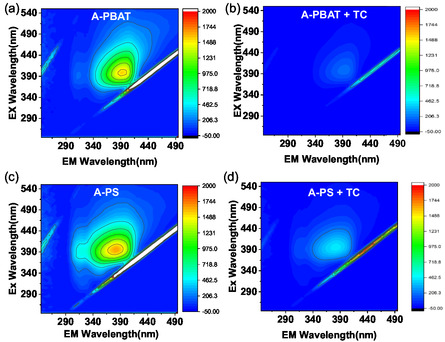
EEM spectra for aged a,b) PBAT and c,d) PS with and without TC.

A 3D‐excitation‐emission matrix spectroscopy (3D‐EEMS) analysis is carried out to investigate the competitive interaction and adsorption of TC on aged MPs. Figure S8(a–b), Supporting Information present the fluorescence characteristics of fresh MPs, where no active zones confirm their pristine nature.^[^ ^58^ ^]^ In contrast, excitation‐emission matrix (EEM) spectra for aged PBAT and PS (**Figure** **6**a,b) show strong fluorescent zones confirming the presence of abundant functional groups, owing to aging in surface water under irradiation. Given that TC is an inactive fluorescent molecule, its interaction with aged MPs likely results in coverage of the aged MPs’ surface, which in turn diminishes fluorescence intensity.^[^ ^59^ ^]^ Figure 6c,d demonstrates that the EEM spectra after TC adsorption exhibit a shift toward the blue region alongside a decrease in intensity confirming a direct interaction between TC and aged MPs. The fluorescence quenching ratio (FQR) is calculated to quantify TC's adsorption efficiency on aged PBAT and PS based on EEMS intensity changes, reflecting the decrease in fluorescence upon adsorption.

$$\text{FQR}=\frac{I_{0}-I}{I_{0}}\times 100\%$$

where *I*_0_ is the initial fluorescence intensity of the aged MPs before TC adsorption and *I* is the fluorescence intensity after adsorption.

Aged PBAT exhibits an 86.14% ± 0.124 fluorescence quenching, surpassing the 73.51% ± 0.235 of aged PS, indicating its superior adsorption for TC. This is due to the oxidative degradation of PBAT, which introduces functional groups that enhance electrostatic interactions and hydrogen bonding with TC.^[^ ^22^, ^36^ ^]^ In contrast, aged PS shows minimal oxidation and relies on weaker hydrophobic interactions and π–π stacking for TC adsorption.^[^ ^60^ ^]^

### 2.4 External Factors Influencing TC Mobility on Aged MPs

The pH‐resolved adsorption–desorption study is critical for forecasting TC mobility on aged MPs.^[^ ^60^ ^]^ To evaluate these dynamics, adsorption experiments were conducted by equilibrating aged PBAT and PS with TC (50 mg L^−1^) across pH 2–10, followed by desorption tests where pH 7‐loaded MPs were transferred to fresh buffers at pH 2, 5, 8, and 10 (Figure S9(a–b), Supporting Information). In the case of the pH effect, under strongly acidic conditions (pH 2), TC predominantly exists as TCH_3_^+^, thus leading to low adsorption (5–7 mg g^−1^) on both PBAT and PS due to electrostatic repulsion with positively charged MPs surfaces. The desorption at pH 2 also reaches 78–86%, reflecting unstable binding (Figure S9(b), Supporting Information). At mildly acidic pH 5, TC adopts a zwitterionic form, enhancing adsorption (15–23 mg g^−1^) through mixed‐charge interactions, though desorption diverges sharply (34% for PBAT vs 53% for PS), suggesting stronger hydrogen bonding or hydrophobic interactions on PBAT. Neutral pH 7 maximizes adsorption (32–35 mg g^−1^) while minimizing desorption (<5%), as TC's neutral form (H_2_TC^0^) facilitates stable hydrophobic binding. In highly alkaline environments (pH 8–10), adsorption declines (24–30 mg g^−1^) due to electrostatic repulsion between negatively charged TC species (HTC^−^, TC^2−^) and microplastic surfaces, with desorption rising notably at pH 10 (25–29%). Crucially, aged PBAT, while outperforming PS in adsorption at pH ≥ 5, exhibits heightened desorption at pH extremes (2 and 10), likely due to its carboxyl‐rich surface, which enhances sorption capacity but also fosters pH‐sensitive electrostatic interactions.^[^ ^52^, ^61^ ^]^ This duality positions biodegradable PBAT as a transient TC sink under neutral conditions but as a potential secondary pollution source in acidic or highly alkaline environments. In contrast, PS, though less adsorptive, demonstrates more stable retention across pH gradients.^[^ ^53^ ^]^

Ionic‐strength suppression is studied to assess the effect of salt on the surface charge and to develop a mechanistic, electrostatic framework for TC adsorption over aged MPs.^[^ ^62^ ^]^ The electrokinetic measurements (Table S8–9 and Figure S10, Supporting Information) further show that adding NaCl or KCl (0–30 mg L^−1^) raises the ionic strength from 0.010 M to ≈0.0105 M (10 mM HEPES), progressively compressing the double layer and unmasking fixed charges on the MPs. Figure S10, Supporting Information shows that fresh MPs start only mildly positive (+5.5 mV for PBAT, +6.9 mV for PS), and once salt is introduced ζ rapidly inverts, ending at −16.5 mV (PBAT) and −19.4 mV (PS) at 30 mg L^−1^ NaCl. In contrast, aged MPs, already rich in carbonyl and hydroxyl groups, begin strongly negative (−52 mV PBAT, −49 mV PS) and are driven only part‐way toward neutrality by the same salt doses, finishing at −22.1 and −17.6 mV, respectively (Figure S10, Supporting Information). Throughout, PBAT remains the more negative, consistent with its larger CI drop, lower contact angle, and denser polar functionality. Table S9, Supporting Information confirms that equal masses of NaCl deliver 15–27% more moles than KCl, shortening κ^−1^ from 3.04 to 2.96 nm vs 2.98 nm. This extra 0.02 nm compression yields stronger charge attenuation. For example, at 20 mg L^−1^ salt, aged PBAT is −28.1 mV in NaCl but −34.3 mV in KCl, an 18% additional neutralization. The steeper ζ decay in NaCl parallels the sharper drop in TC uptake (Figure 5), confirming that ion‐specific charge screening governs the electrostatic component of adsorption rather than ionic size.^[^ ^21^, ^25^, ^57^ ^]^ Thus, Na‐rich saline waters will more efficiently mobilize TC from aged PBAT and PS than K‐dominated waters, with PBAT remaining the more interactive sorbent because of its higher residual negative charge and greater density of photo‐generated polar groups.

## 3 Conclusion

In conclusion, this study shows that PBAT and PS, which commonly coexist as suspended debris, have significantly different photodegradation dynamics under submerged conditions. UV‐induced aging in surface water significantly alters the adsorption behavior of both MPs, with PBAT undergoing substantial oxidative transformation, marked by a 16% decline in carbonyl index, a 55 mV shift in ζ‐potential, and enhanced surface functionalization with hydroxyl, carbonyl, and carboxyl groups. These structural changes amplify its adsorption capacity for TC, doubling its Langmuir capacity (*q*_m_ ≈ 101 mg g^−1^) and tripling its binding kinetics, while fluorescence quenching (86%) underscores robust sorbate affinity. In contrast, PS exhibits minimal oxidation (6% CI reduction) and retains a hydrophobic surface, relying on π–π interactions and van der Waals forces for TC adsorption (*q*_m_ ≈ 98 mg g^−1^), which is more susceptible to ionic interference. Both MPs interact with TC maximally at pH 7, yet alkaline water releases 78% of the TC from PBAT against 62% from PS, underscoring PBAT's greater density of deprotonated sites. Ionic strength further modulates adsorption, with Na^+^ suppressing uptake more effectively than K^+^, though PBAT maintains superior performance. Collectively, these results indicate that aged PBAT acts as a short‐term, highly reactive sink, and, under alkaline or saline shifts, a secondary source of antibiotics, whereas aged PS, with slower surface evolution, functions chiefly as a long‐range transport vector. Accordingly, management efforts should prioritize the removal or stabilization of fine PBAT fragments in wastewater streams and incorporate UV‐resistance or controlled‐degradation additives into biodegradable plastic formulations to curb episodic pollutant release.

## 4 Experimental Section

4.1

4.1.1

##### Materials and Reagents

PS and PBAT MPs, averaging 50 μm in size, were bought from Mingshuo Chemical Co., Ltd. TC, HCl, ethanol, NaOH, and additional reagents were acquired from Sino Pharm Chemical Reagent Co., Ltd. All organic reagents used for high performance liquid chromatography (HPLC) were chromatographically pure. Acetonitrile (99.9%) from Sigma‐Aldrich Co. Ltd. (Shanghai, China) and oxalic acid and phosphoric acid were used. Shanghai Macklin Biochemical Co., Ltd. supplied analytical grade (AR) methanol and isopropanol. All solutions were prepared using ultrapure water.

##### Photoaging of PBAT and PS MPs in Surface Water

Photoaging of MPs (average size 50 μm) was conducted in a controlled environment to simulate natural UV exposure in surface waters. Fresh MPs were submerged in collected river water (Table S1, Supporting Information for water characteristics) to replicate environmental conditions. The MPs were exposed to ultraviolet A (UVA)‐340 nm light, calibrated using a light intensity calibrator (ILT 2400), and kept at 10 cm elevation (2 × 40 W, irradiance 60 mW cm^−2^) for 30 days using a UV aging box (ZT‐UV‐50SUV‐Guangdong Zhong tian Instrument Co., Ltd). A 30‐day exposure period was selected to allow for sufficient photoaging of both PBAT and PS. PBAT, being biodegradable, degrades quickly, while PS, a nondegradable polymer, takes longer to show significant aging. Thus, this timeframe ensures both polymers undergo comparable aging for meaningful comparison. MPs were turned over every 24 h to ensure uniform exposure. In this case, high UV irradiance was used to compensate for light attenuation from surface water.^[^ ^22^ ^]^ A higher concentration of MPs (1 g L^−1^) was chosen to reflect contamination levels in the environment, while a high TC concentration (10 mg L^−1^) ensured measurable adsorption interactions.^[^ ^63^ ^]^ The hydration levels were controlled, with evaporated water replenished every 48 h to maintain consistent volume. The weathering chamber maintained ambient conditions of 25 ± 1 °C and 50 ± 5% relative humidity (RH), mimicking real environmental conditions.^[^ ^64^, ^65^ ^]^

##### Characterizations

The MP's surface morphology was ascertained using an SEM (Nova Nano SEM 450, USA) coupled with energy‐dispersive X‐ray spectroscopy or EDX for elemental analysis. An 3D‐EEM analysis was conducted to confirm the alterations in the interactional behavior of MPs. The characterization of surface morphologies, functional groups, and crystal structures was accomplished through FTIR (Vertex 70, Bruker), XRD (D8 Advance, Bruker), and XPS (Escalab 250Xi, Thermo Fisher Scientific). Additionally, the wettability of polymer surfaces subjected to UV radiation at room temperature was measured using the OCA 15EC goniometer tilting unit TBU 100 (Data Physics Instruments GmbH, Filderstadt, Germany). Contact angle measurements were conducted using a Krüss Drop Shape Analyzer (DSA) (CAM, JY‐82B, Germany), with triplicate measurements per sample. The mean contact angle and standard deviation (±SD) were calculated and reported to ensure precision and reproducibility. The Zeta sizer Nano ZS (ZEN3600UK) was employed to measure the surface potential, with triplicate measurements taken for each sample. Error margins (±SD) were reported to reflect the measurement variability. TC concentrations were analyzed using reversed‐phase HPLC with columns such as the Shim‐pack GIS C18 column (4.60 × 250.00 mm, 5.00 μm) and L1 column (3 μm, 150 × 4.6 mm)^[^ ^45^ ^]^ with data recorded in triplicates and with error margins reported as ±SD.

##### Statistical Analyses

All experimental measurements were performed in triplicate and are reported as mean ± standard deviation (SD), with error bars representing variability. Prior to hypothesis testing, data normality, and homoscedasticity were assessed using the Shapiro–Wilk and Levene tests, respectively (IBM SPSS Statistics 26; *p* > 0.05 for all datasets). Treatment effects (pH and ionic strength) were evaluated using one‐way analysis of variance (ANOVA); significant differences (*p* < 0.05) were followed by Tukey's honest significant difference (HSD) post‐hoc test. Tukey's critical spacing was calculated using the following equation:

$$\text{HSD}=q_{\alpha ,k,N-K}=\sqrt{\frac{\text{MSE}}{n}}$$

where *q*_*α,k*_, _*N−k*_ is the studentized range statistic at significance level *α*, *k* is the number of groups, *N* is the total number of points used, MSE is the mean square error from the ANOVA, and *n* is the number of replicates per group. For kinetic and isotherm models, adsorption data were fitted to the PFO and PSO models using nonlinear least‐squares regression. The fit quality was assessed by adjusted *R* ^2^ values, RSS, and information criteria such as the AIC and BIC. The coefficients of variation (CV) for raw replicate measurements were <5%, ensuring high analytical precision and consistency across the experiments.

##### Batch Experiments

##### Batch Experiments: Adsorption of TC over Aged MPs

The adsorption kinetics of TC on fresh and aged MPs (PBAT and PS) were investigated under controlled conditions. For each experiment, 20.0 ± 0.1 mg of MPs were added to 20 mL of TC solution (10 mg L^−1^ initial concentration) in 50 mL amber glass centrifuge tubes to prevent photodegradation.^[^ ^66^, ^67^ ^]^ The suspensions were agitated at 150 ± 2 rpm (25 ± 1 °C) using an orbital shaker. Sampling was performed at predetermined intervals (0.5, 1, 3, 6, 12, 24, 48, 96, and 120 h), with 1 mL aliquots withdrawn at each time point and immediately filtered through 0.22 μm nylon membranes (pre‐rinsed with methanol and ultrapure water). The filtrate was analyzed by HPLC (Shimadzu LC‐20AT) equipped with a C18 column (4.6 × 250 mm, 5 μm) using a mobile phase of 0.01 M oxalic acid: acetonitrile (75:25 v/v) at 1.0 mL min^−1^ flow rate. TC quantification was performed at 355 nm wavelength, with calibration curves (*R* ^2^ > 0.999) verified every 20 samples. Adsorption isotherms were determined by exposing 20.0 ± 0.1 mg MPs to 20 mL TC solutions with increasing concentration gradient (2.5, 5, 10, 20, 30, 40, and 50 mg L^−1^) in 50 mL amber tubes. After 48 h equilibration (150 rpm, 25 °C) confirmed by preliminary kinetic studies ‐ samples were processed. For the effect of pH during the adsorption‐desorption test, the bulk TC solutions were first adjusted to the target pH (2, 3, 5, 7, 8, 10) with 0.1 M/NaOH, and the pH was verified at both the start and end of equilibration. In the case of desorption, MPs pre‐equilibrated at pH 7 were removed, and transferred into fresh buffer solutions that had been pre‐adjusted to pH desired pH.^[^ ^68^ ^]^ For the salinity studies, NaCl and KCl were added in concentrations ranging from 5% to 30% (w/v) to achieve varying ionic strengths, representing a gradient from low salinity to brackish condition.^[^ ^69^ ^]^ The adsorption kinetics and modeling were conducted by fitting the experimental data to nonlinear PFO and PSO kinetic and Langmuir and Freundlich isotherm models (Section 2.0, Supporting Information).

## Conflict of Interest

The authors declare no conflict of interest.

## Supporting information

Supplementary Material

## Data Availability

The data that support the findings of this study are available from the corresponding author upon reasonable request.
