# Supplementary material for: Comparative Photo‐Induced Aging of Poly(Butylene Adipate‐co‐Terephthalate) and Polystyrene Microplastics and their Divergent Affinities for Tetracycline in Aquatic Environments
Source: ChemistryOpen. 2025 Jun 25;14(11):e202500243. doi: 10.1002/open.202500243 (PMC12598813; doi:10.1002/open.202500243)
Supplement: Supplementary file 1 — Supplementary Material [file OPEN-14-e202500243-s001.pdf]

# Supplementary Data

## Comparative Photoinduced Aging of PBAT and PS Microplastics and Their Divergent Affinities for Tetracycline in Aquatic Environments

Contents


1. Characteristics of surface water ........ 1

2. Modelling and statistical evaluation of adsorption data ........ 2

2.1 Kinetic Models ........ 1

2.2 Isotherm model ........ 3

2.4 Information criteria (AIC, BIC) ........ 4

3. Desorption ........ 4


**1. Characteristics of surface water**

Water samples were obtained from many locations along a designated river in China to facilitate the decomposition of biodegradable plastics. Table S1 delineates the physical properties of the river water. Fluorescent dissolved organic matter (FDOM) was quantified

using fluorescence spectroscopy, a quick, non-destructive method noted for its excellent sensitivity, selectivity, cost-effectiveness, and minimal sample preparation requirements[1].

## 2. Modelling and statistical evaluation of adsorption data

### 2.1 Kinetic models

Pseudo-first-order (PFO) and pseudo-second-order (PSO) equations were applied to discriminate whether TC uptake on the aged and fresh microplastics is governed primarily by rapid film diffusion (PFO) or by slower surface-reaction/chemisorption steps (PSO), thereby linking rate constants to the extent of photo-induced functionalization[2].

**Pseudo-first-order:** $ln(q_e - q_t) = lnq_e - k_1\, t$

Here, $q_t$ and $q_e$ are the amounts adsorbed (mg/g) at time ($t$) and equilibrium, respectively.

**Pseudo-second-order:** $\frac{t}{q_t} = \frac{1}{k_2 (q_e)^2} + \frac{t}{q_e}$

Here, $k_1$ is the pseudo - first - order rate constant (1/time), and $t$ is contact time, $k_2$ is the pseudo - second - order rate constant (g/mg.h)

**Pseudo-first-order Prediction:** $qt = qe(1 - \exp(-k_1 t)$

Here, $q_t$ is the adsorption capacity (mg/g) at time, $q_e$ is equilibrium adsorption capacity, maximum adsorption), $k_1$is the rate constant ($h^{-1}$), and $t$ is the time at equilibrium[3].

**Pseudo-second-order Prediction:** $q_t \; = \frac{q_e}{1 + k_2\, q_e t}$

Here, $q_e$ is the equilibrium adsorption capacity (mg/g), $k_1$is the rate constant ($h^{-1}$), $t$ is the time at equilibrium.

## 2.2 Adsorption model

Langmuir and Freundlich's plots were employed to determine whether TC binds as a uniform monolayer to a finite set of sites (Langmuir) or distributes heterogeneously over energetically diverse surfaces (Freundlich), information essential for comparing the sorptive heterogeneity created by submerged UV aging[4].

**Freundlich model:** $q_e = K_f \times C_e^{\frac{1}{n}}$

Here $q_e$ is the amount of adsorbate (mg/g), $C_e$ is a solution at equilibrium (mg/ L), $K_f$ (mg/g)(L/mg)*1/n*) *i*s the Freundlich constant, and $n$ is the dimensionless Freundlich exponent[5].

**Langmuir model:** $q_e = \frac{q_m \times K_L C_e}{1+K_L C_e}$

Here,$q_m$ is the maximum (monolayer) adsorption capacity (mg/g), $K_L$ is the Langmuir constant (L/mg), and $C_e$ (mg/ L) is the equilibrium solution concentration[5].

## 2.3 Residual Calculation

Point-by-point residuals were computed to visualize systematic deviations between models and data, revealing any time or concentration domains where the chosen kinetic or isotherm expression fails to describe the process accurately[6].

**Residuals:** $e_i = q_{t,}exp - q_t pred$

**Here,** $e_i$ is the residual, $q_t$, *exp* is the observed adsorption capacity (at 120 hours), $q_t$, *pred* is the predicted adsorption capacity based on the model.

## 2.4 Information criteria (AIC, BIC)

Akaike and Bayesian information criteria were calculated to rank competing kinetic or isotherm models objectively, penalizing over-parameterization and thus identifying the mechanistically most plausible equation for each polymer–antibiotic system[7].

$$AIC = nln\left(\frac{RSS}{n}\right) + 2k$$

$$BIC = nln\left(\frac{RSS}{n}\right) + kln(n)$$

Here, $n$ is the number of materials, $k$ is a parameter of models like $k_1$ and $k_2$

## 2.5 Residual sum of square (RSS)

The RSS provides a quantitative measure of absolute misfit on which the information criteria are based; smaller RSS values indicate closer agreement between predicted and observed adsorption capacities and, by extension, a more credible representation of the underlying sorption mechanism[7].

$$RSS = \sum_{i=1}^{n}(ei)^2$$

## 3. Desorption

Adsorption was first carried out by contacting 10 mg of MPs with 50 mL of a 50 mg $L^{-1}$ tetracycline (TC) solution (10 mM HEPES, pH 7.0, 25 °C) for 120 h. After equilibrium, the suspension was centrifuged (5 000 g, 10 min); the supernatant was removed, the particles were rinsed twice with deionized water, and an equal volume (50 mL) of fresh background solution adjusted to the desired pH (2, 4, 6, 7, 8, 10; 10 mM HEPES) was

added. The mixture was then agitated at 150 rpm and 25 °C for a further 120 h to allow desorption[8]. Following centrifugation, the TC concentration in the supernatant was quantified by HPLC.:

$$\boldsymbol{Desorption\ percentage}\ (\%) = \frac{C_{des} \times V}{q_{ads} \times m} \times 100$$

where $C_{des}$ (mg $L^{-1}$) is the TC concentration after desorption, V (L) is the solution volume, $q_{ads}$ (mg $g^{-1}$) is the amount initially adsorbed during the preceding uptake stage, and m (g) is the mass of MPs. All experiments were performed in triplicate, and results are reported as mean ± SD.

**Figure S1**

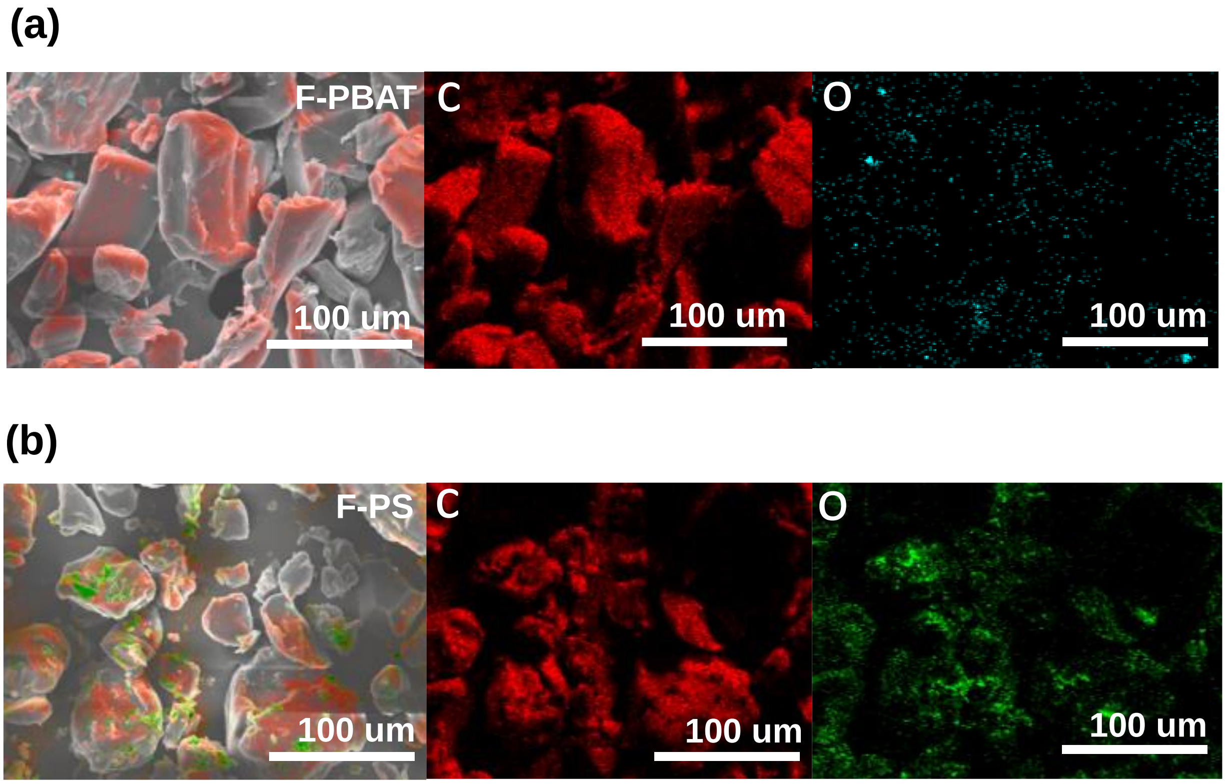


**Figure S1** EDS mapping of fresh PBAT (a) and fresh PS (b) confirming presence of C., and O as major elements.

# Figure S2

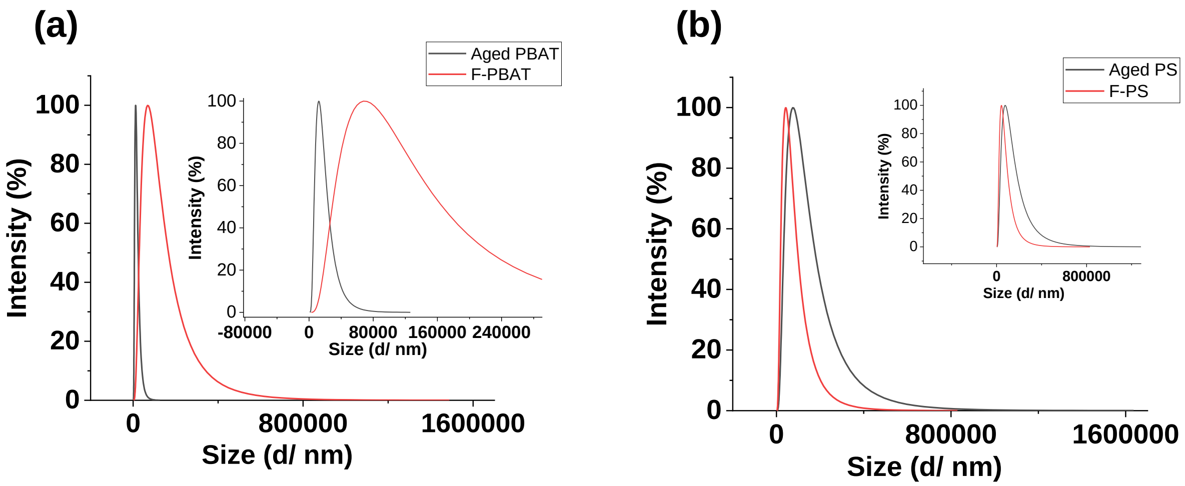


**Figure S2** Particle size distribution of fresh and aged (a) PBAT and (b) PS.

**Figure S3**

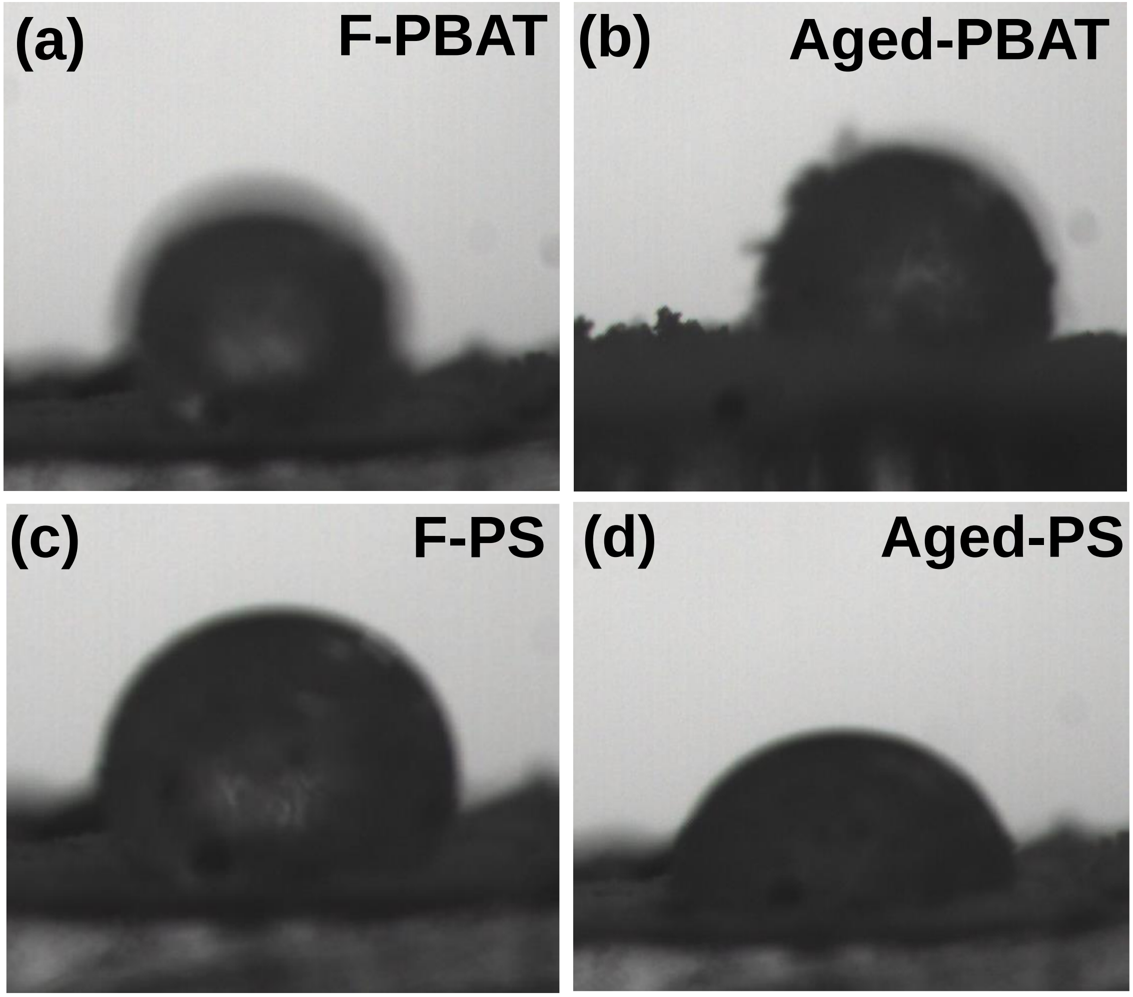


**Figure S3** contact angle measurements of (a) fresh PBAT, (b) aged PBAT, (c) fresh PS, and (d) aged PS, illustrating the changes in surface wettability over time.

**Figure S4**

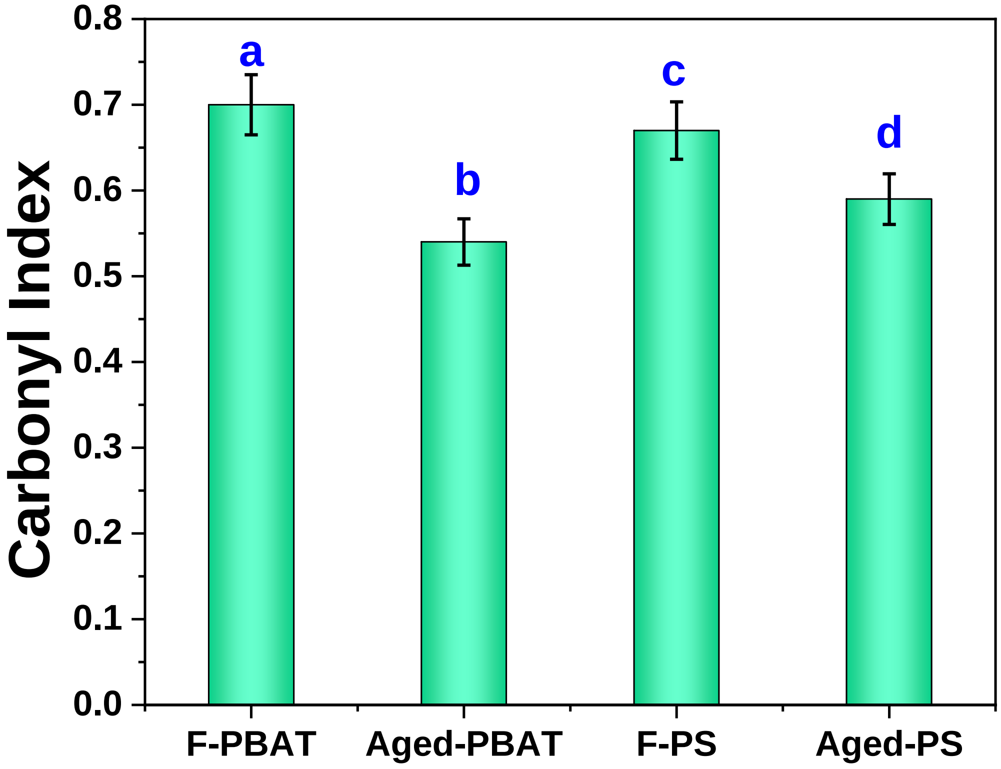


**Figure S4** change in CI index of fresh and aged PBAT and PS. Bars represent mean ± SD (n = 3) and letters above bars denote statistical grouping: columns that share a letter do not differ significantly (one-way ANOVA followed by Tukey's HSD, $p \geq 0.05$).

**Figure S5**

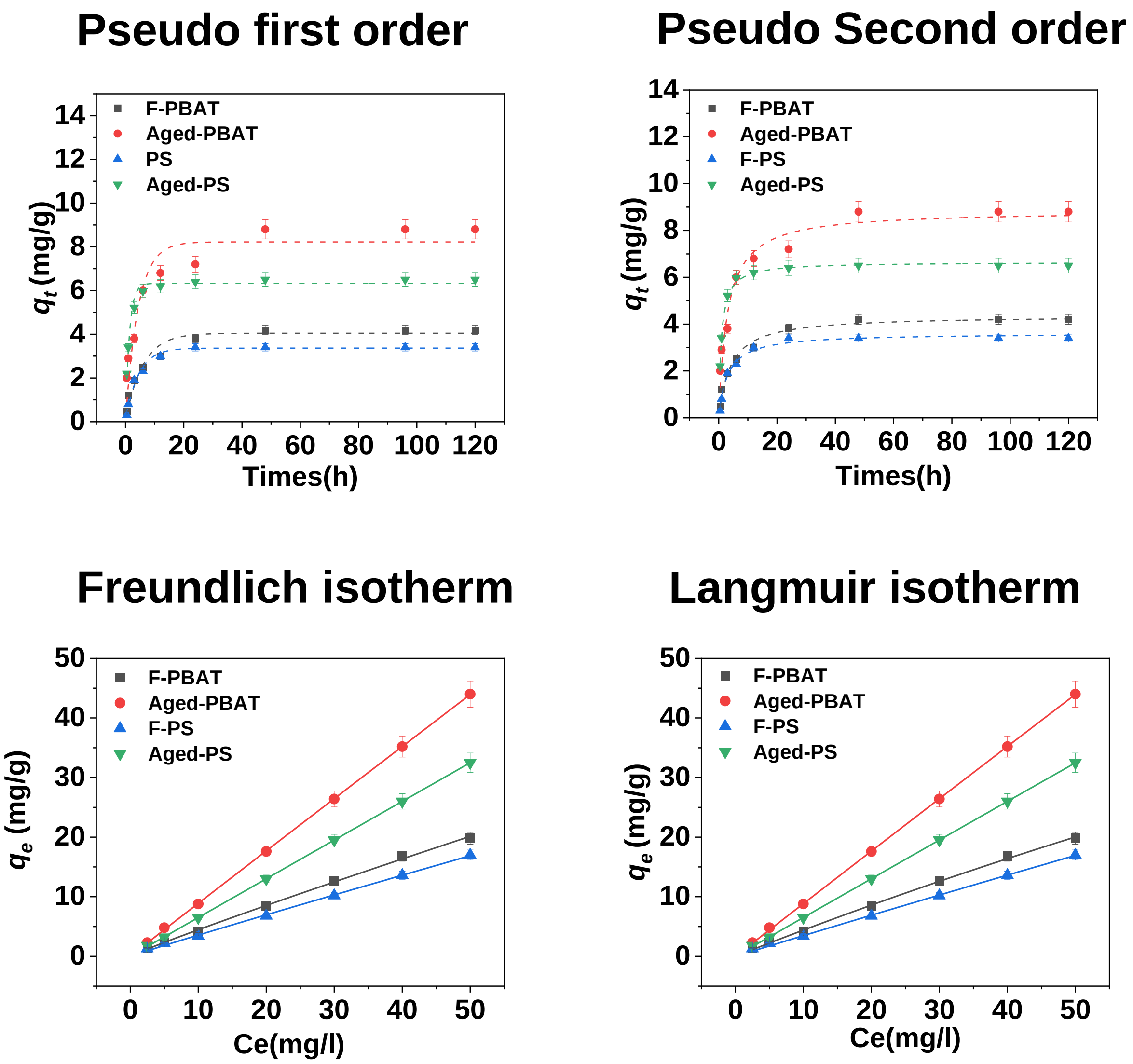


**Figure S5** Kinetic Fitting parameters for Pseudo first and second order model, and Freundlich and Langmuir fittings for fresh and aged PBAT and PS MPs.

**Figure S6**

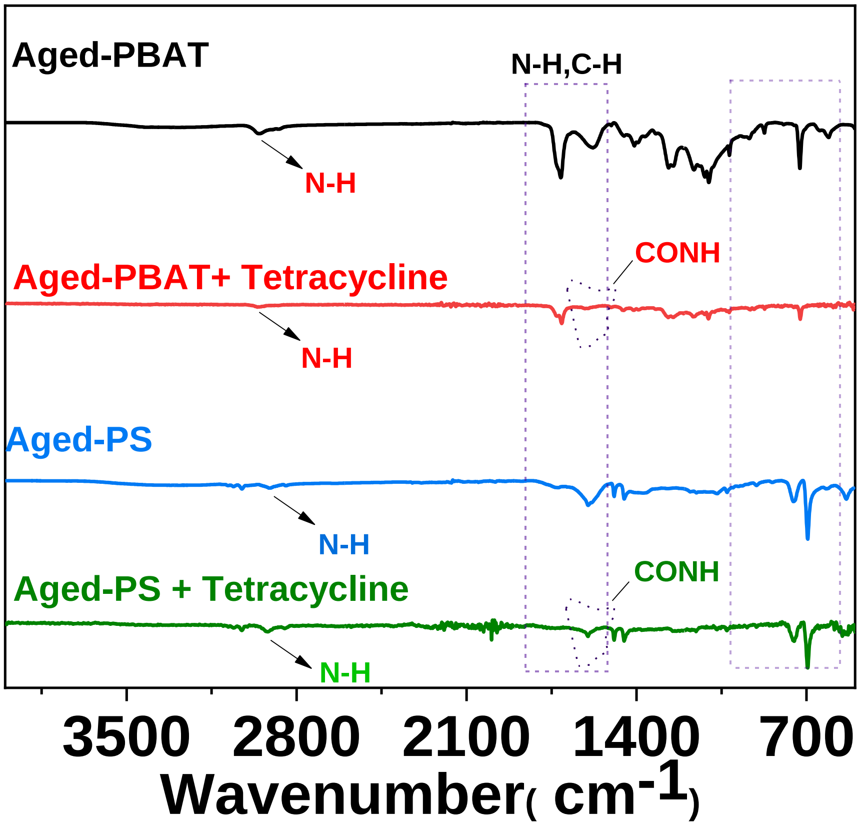


**Figure S6** FTIR spectra for aged PBAT and PS showing shift in typical functional groups post TC adsorption.

## Figure S7

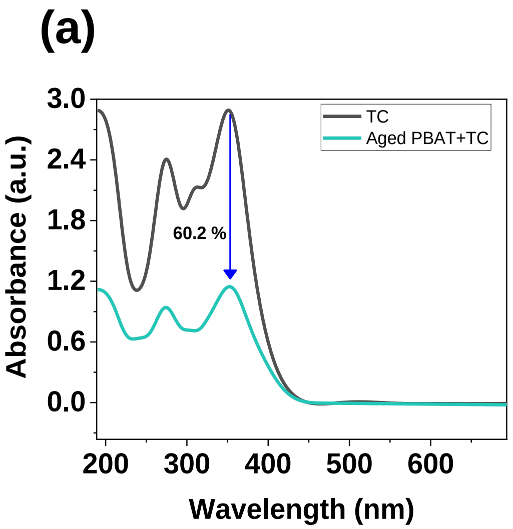


**Figure S7** UV-Vis spectral profile for TC using (a) aged PBAT and (b) aged PS.

## Figure S8

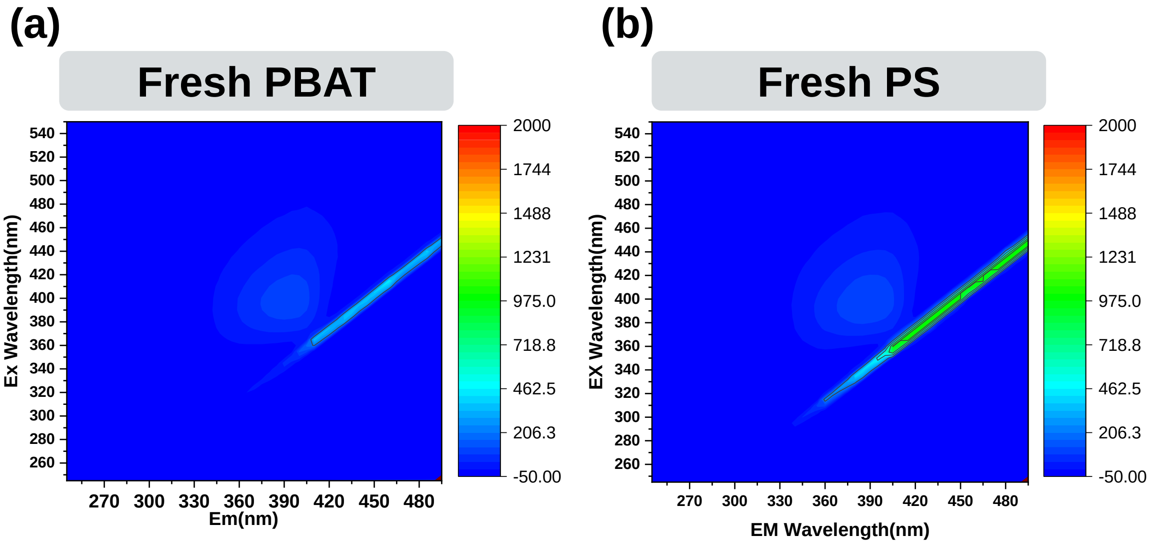


**Figure S8** EMMS spectra of fresh (a) PBAT and (b) PS.

## Figure S9

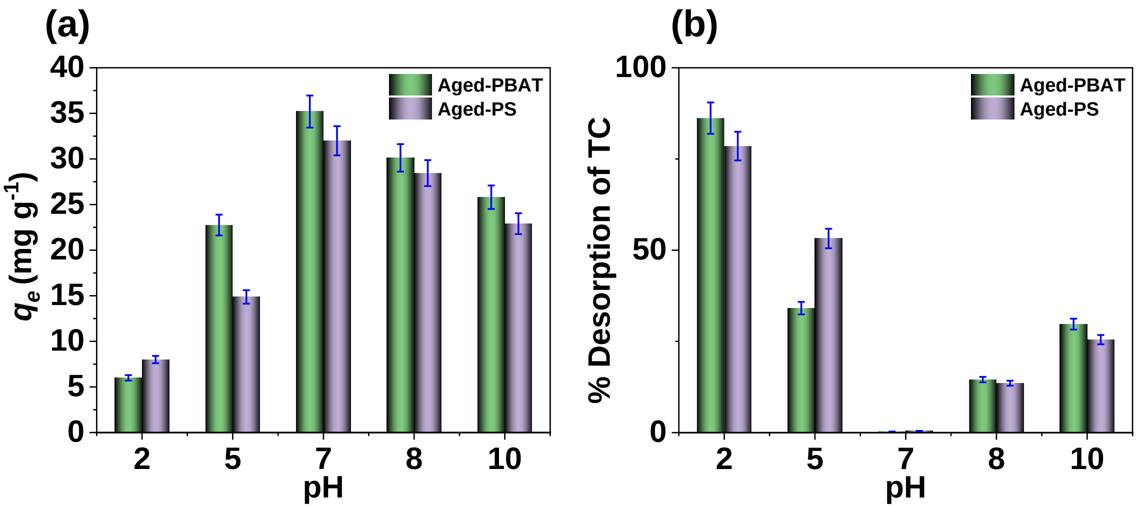


**Figure S9** (a) variation in adsorption capacity ($Q_m$) of TC over aged MPs with change in pH, and (b) % desorption of TC over aged MPs. average values (n = 3), with error bars indicating that coefficients of variation equal to <5%.

## Figure S10

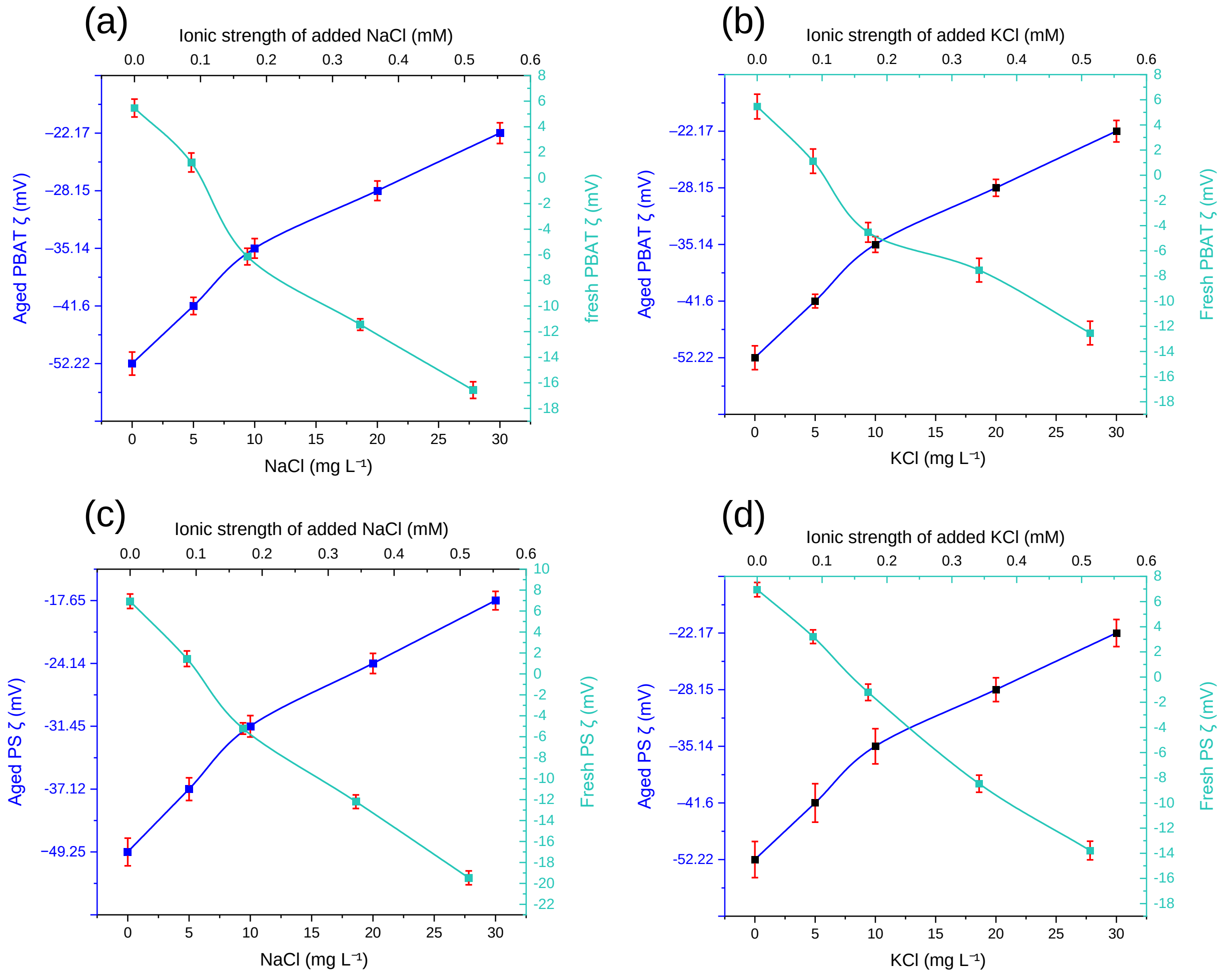


**Figure S10** (a, b) Influence of incremental NaCl and KCl on ζ-potential of fresh and UV-aged PBAT and (c, d) PS at pH 7 using 10 mM HEPES, 25 °C as buffer electrolyte.

**Table S1.** Surface water quality parameters.

| Index | Range ( ) | Mean | Standard deviation |
|---|---|---|---|
| Temperature (°C) | 24.4-27.9 | 26.2 | 1.77 |
| pH | 6.98-7.30 | 7.14 | 0.16 |
| Salinity (PSU) | 3.12-4.13 | 3.63 | 0.50 |
| Fluorescent dissolved organic matter (RFU) | 5.38-14.9 | 10.1 | 4.78 |
| Biochemical oxygen demand (mg/L) | 2.76-4.12 | 3.44 | 0.68 |
| Chemical oxygen demand (mg/L) | 1.95-2.76 | 2.36 | 0.40 |
| Dissolved oxygen (mg/L) | 8.74-11.5 | 10.14 | 1.39 |
| Turbidity | 25.3-41.7 | 33.54 | 8.19 |

**Table S2.** Concentrations of common ions in surface water.

| Ions | Concentrations (mmol $L^{-1}$) | Standard deviation |
|---|---|---|
| $Ca^{2+}$ | 0.930 | 0.020 |
| $Mg^{2+}$ | 0.512 | 0.014 |
| $Na^{+}$ | 0.30 | 0.016 |
| $K^{+}$ | 0.06 | 0.012 |
| $F^{-}$ | 0.01 | 0.016 |
| $Cl^{-}$ | 0.15 | 0.018 |
| $NO_3$ | 0.09 | 0.019 |
| $SO_4^{2-}$ | 0.49 | 0.203 |
| $HCO_3^{-}$ | 2.00 | 0.023 |

**Table S3.** Parameters for Kinetics of adsorption characteristics for fresh and aged MPs

| **Pseudo-first order** | **F-PBAT** | | | **Aged-PBAT** | | | **F-PS** | | | **Aged-PS** | | |
|---|---|---|---|---|---|---|---|---|---|---|---|---|
| | $R^2$ | $q_e$ (mg/g) | $k_1$ ($h^{-1}$) | $R^2$ | $q_e$ (mg/g) | $k_1$ ($h^{-1}$) | $R^2$ | $q_e$ (mg/g) | $k_1$ ($h^{-1}$) | $R^2$ | $q_e$ (mg/g) | $k_1$ ($h^{-1}$) |
| Tetracycline | 0.94 | 4.04 | 0.169 | 0.88 | 8.22 | 0.22 | 0.98 | 3.36 | 0.22 | 0.97 | 6.32 | 0.72 |

| **Pseudo-second order** | $R^2$ | $q_e$ (mg/g) | $k_2$ ( g/mg·h) | $R^2$ | $q_e$ (mg/g) | $k_2$ (g/mg·h) | $R^2$ | $q_e$ (mg/g) | $k_2$ (g/mg·h) | $R^2$ | $q_e$ (mg/g) | $k_2$ ( g/mg·h) |
|---|---|---|---|---|---|---|---|---|---|---|---|---|
| Tetracycline | 0.98 | 4.36 | 0.057 | 0.95 | 8.84 | 0.037 | 0.98 | 3.61 | 0.09 | 0.99 | 6.65 | 0.163 |

**Table S4.** Parameters for adsorption isotherms of fresh and aged MPs.

| **Freundlich Isotherm** | **Tetracycline** | | |
|---|---|---|---|
| | ***1/n*** | $K_f$ **((mg/g) (L/mg)1/n))** | $R^2$ |
| **F-PBAT** | 0.93 | 0.51 | 0.99 |
| **Aged-PBAT** | 0.99 | 0.90 | 0.99 |
| **F-PS** | 0.96 | 0.38 | 0.99 |
| **Aged-PS** | 0.99 | 0.65 | 0.99 |
| **Langmuir Isotherm** | **Tetracycline** | | |
| | $q_m$ **(mg/g)** | $K_L$ **(L/mg)** | $R^2$ |
| **F-PBAT** | 23.59 | 0.0278 | 0.99 |
| **Aged-PBAT** | 101.0 | 0.0133 | 0.99 |
| **F-PS** | 60.34 | 0.0091 | 0.99 |
| **Aged-PS** | 98.00 | 0.0095 | 0.99 |

**Table S5.** Observed and predicted adsorption capacities (PFO) model for F-PBAT, A-PBAT, F-PS, and A-PS at different time points. Residuals represent the difference between observed and predicted values.

| Time (h) | F-PBAT Observed | F-PBAT (PFO Predicted) | Residuals (PFO) | Aged-PBAT (Observed) | Aged-PBAT (PFO Predicted) | Residuals (PFO) | F-PS (Observed) | F-PS (PFO Predicted) | Residuals (PFO) | Aged-PS (Observed) | Aged-PS (PFO Predicted) | Residuals (PFO) |
|---|---|---|---|---|---|---|---|---|---|---|---|---|
| 0.5 | 0.48 | 2.18 | -1.62 | 2 | 4.64 | -1.64 | 0.3 | 1.79 | -1.49 | 2.2 | 3.82 | -1.62 |
| 1 | 1.2 | 2.27 | -0.97 | 2.9 | 4.88 | -1.98 | 0.8 | 1.88 | -1.08 | 3.4 | 4.37 | -0.47 |
| 3 | 1.9 | 2.62 | -0.72 | 3.8 | 5.80 | -1 | 1.9 | 2.24 | -0.34 | 5.2 | 5.82 | -0.02 |
| 6 | 2.5 | 3.08 | -0.58 | 6 | 6.94 | -0.94 | 2.3 | 2.68 | -0.38 | 5.9 | 6.41 | -0.41 |
| 12 | 3 | 3.71 | -0.71 | 6.8 | 8.21 | -1.41 | 3 | 3.17 | -0.17 | 6.2 | 6.49 | -0.29 |
| 24 | 3.8 | 4.12 | -0.32 | 7.2 | 8.75 | -1.5 | 3.4 | 3.38 | 0.01 | 6.4 | 6.5 | -0.10 |
| 48 | 4.2 | 4.19 | 0 | 8.8 | 8.79 | 0 | 3.4 | 3.39 | 0 | 6.5 | 6.5 | 0 |
| 96 | 4.2 | 4.21 | 0 | 8.8 | 8.89 | 0 | 3.4 | 3.4 | 0 | 6.5 | 6.5 | 0 |
| 120 | 4.2 | 4.21 | 0 | 8.8 | 8.89 | 0 | 3.4 | 3.4 | 0 | 6.5 | 6.5 | 0 |

**Table S6.** Observed and predicted adsorption capacities PSO) model for F-PBAT, A-PBAT, F-PS, and A-PS at different time points. Residuals represent the difference between observed and predicted values.

| Time (h) | F-PBAT Observed | F-PBAT (PSO Predicted) | Residuals (PSO) | Aged-PBAT (Observed) | Aged-PBAT (PSO Predicted) | Residuals (PSO) | F-PS (Observed) | F-PS (PSO Predicted) | Residuals (PSO) | Aged-PS Observed | Aged-PS (PSO Predicted) | Residuals (PSO) |
|---|---|---|---|---|---|---|---|---|---|---|---|---|

| | | | | | | | | | | | | |
|---|---|---|---|---|---|---|---|---|---|---|---|---|
| 0.5 | 0.48 | 4.08 | -3.81 | 2 | 8.64 | -6.64 | 0.3 | 3.25 | -2.95 | 2.2 | 6.01 | -3.81 |
| 1 | 1.2 | 3.97 | -2.18 | 2.9 | 8.48 | -5.58 | 0.8 | 3.11 | -2.31 | 3.4 | 5.58 | -2.18 |
| 3 | 1.9 | 3.58 | 0.85 | 3.8 | 7.92 | -4.12 | 1.9 | 2.67 | -0.77 | 5.22 | 4.36 | 0.85 |
| 6 | 2.5 | 3.12 | 2.69 | 6 | 7.20 | -1.20 | 2.3 | 2.20 | 0.09 | 5.98 | 3.28 | 2.69 |
| 12 | 3 | 2.49 | 4.00 | 6.8 | 6.09 | 0.70 | 3 | 1.63 | 1.36 | 6.2 | 2.19 | 4.00 |
| 24 | 3.8 | 1.77 | 5.07 | 7.2 | 4.66 | 2.53 | 3.4 | 1.07 | 2.32 | 6.4 | 1.32 | 5.07 |
| 48 | 4.2 | 1.12 | 5.76 | 8.8 | 3.17 | 5.62 | 3.4 | 0.63 | 2.76 | 6.5 | 0.73 | 5.76 |
| 96 | 4.2 | 0.64 | 6.10 | 8.8 | 1.93 | 6.86 | 3.4 | 0.35 | 3.04 | 6.5 | 0.39 | 6.10 |
| 120 | 4.2 | 0.53 | 6.18 | 8.8 | 1.61 | 7.18 | 3.4 | 0.28 | 3.11 | 6.5 | 0.31 | 6.18 |

**Table S7.** AIC, BIC, and RSS values for the PFO and PSO models for F-PBAT, A-PBAT, F-PS, and A-PS. AIC and BIC represent the model selection criteria, while RSS shows the residual sum of squares for each model.

| **Material** | **AIC (PFO)** | **BIC (PFO)** | **AIC (PSO)** | **BIC (PSO)** | **Material** | **RSS (PFO)** | **RSS (PSO)** |
|---|---|---|---|---|---|---|---|
| F-PBAT | -0.43 | -0.04 | 21.63 | 22.02 | F-PBAT | 5.49 | 63.84 |
| Aged-PBAT | 11.26 | 11.6 | 33.21 | 33.60 | Aged-PBAT | 20.18 | 231.18 |
| F-PS | -4.058 | -3.66 | 19.18 | 19.58 | F-PS | 3.67 | 48.65 |
| Aged-PS | -2.639 | -2.24 | 30.84 | 31.23 | Aged-PS | 4.30 | 177.66 |

**Table S8**. ζ-potential (mV) of fresh and UV-aged PBAT and PS as a function of added NaCl or KCl (0–30 mg $L^{-1}$; 10 mM HEPES, pH 7.0, 25 °C). Values are mean ± SD (n = 3).

| Added salt (mg $L^{-1}$) | Added salt (mM) | ζ-potential (mV) | | | | ζ-potential (mV) | | | |
|---|---|---|---|---|---|---|---|---|---|
| | | Aged PBAT | | Fresh PBAT | | Aged PS | | Fresh PS | |
| | | NaCl | KCl | NaCl | KCl | NaCl | KCl | NaCl | KCl |
| 0 ‡ | 0.000 | -52.2±1.21 | -52.2±2.65 | 5.46±1.21 | 5.46±1.92 | −49.2±3.21 | −49.2±1.98 | 6.94±3.45 | 6.95±1.99 |
| 5 | 0.086 | -41.6±1.15 | -42.6±3.12 | 1.21±2.41 | 1.12±2.12 | -37.1±1.26 | -40.2±1.76 | 1.45±3.11 | 3.21±2.45 |
| 10 | 0.171 | -35.1±3.45 | -38.7±1.95 | -6.14±3.12 | -4.54±1.23 | -31.4±3.03 | -35.1±3.13 | -5.21±1.97 | -1.21±2.98 |
| 20 | 0.342 | -28.1±2.75 | -34.2±2.61 | -11.4±3.35 | -7.54±1.21 | -24.1±3.12 | -29.1±3.12 | -12.2±2.65 | -8.47±1.47 |
| 30 | 0.513 | -22.1±2.14 | -30.1±1.83 | -16.5±3.19 | -12.5±2.22 | -17.6±1.10 | -25.7±2.91 | -19.4±1.22 | -13.7±3.71 |

‡ Baseline 10 mM HEPES; no chloride salt added.

**Table S9.** Incremental ionic strength (I) and Debye length ($\kappa^{-1}$) generated by the NaCl and KCl additions used in Table S9. Baseline I = 0.010 M from 10 mM HEPES; $\kappa^{-1}$ calculated with $\kappa^{-1}$ (nm)= 0.304 / √ I (M) for a 1:1 electrolyte at 25 °C.

| Added NaCl (mg $L^{-1}$) | Added NaCl (mmol $L^{-1}$) | Total ionic strength, I (M)* | $\kappa^{-1}$ (nm)† |
|---|---|---|---|
| 0 ‡ | 0.000 | 0.010000 | 3.04 |
| 5 | 0.0856 | 0.010086 | 3.03 |
| 10 | 0.171 | 0.010171 | 3.01 |
| 20 | 0.342 | 0.010342 | 2.99 |
| 30 | 0.513 | 0.010513 | 2.96 |

| Added KCl (mg $L^{-1}$) | Added KCl (mmol $L^{-1}$) | Total ionic strength, I (M)* | $\kappa^{-1}$ (nm)† |
|---|---|---|---|

| | | | |
|---|---|---|---|
| 0 ‡ | 0.000 | 0.010000 | 3.04 |
| 5 | 0.0671 | 0.010067 | 3.03 |
| 10 | 0.134 | 0.010134 | 3.02 |
| 20 | 0.268 | 0.010268 | 3.00 |
| 30 | 0.402 | 0.010402 | 2.98 |

* Total ionic strength = 0.010 M from the 10 mM HEPES buffer plus the listed NaCl or KCl contribution.
† Debye length calculated with $\kappa^{-1}$ (nm) = 0.304 / $\sqrt{I}$ (M) for a 1:1 monovalent electrolyte at 25 °C.
‡ Baseline buffer only; no chloride salt added.

## 4. References


1. Wu, C., J. Wan, J. Wang, J. Cai, X. Ren, Y. Wang, and Z. Bi, *Three-dimensional Fluorescence Spectrum Characteristics and Source Analysis of Dissolved Organic Matter in Sewage Outfall into the Sea of Dongguan.* Water, Air, & Soil Pollution, 2025. **236**(1): p. 1-15.
2. Assafi, A., L. Hejji, Y.A.E.H. Ali, N.B. Seddik, L. Pérez-Villarejo, P.J. Sánchez-Soto, B. Souhail, and A. Azzouz, *The utility of L-cysteine-functionalized graphene oxide for the adsorptive removal of 4-nitrophenol from environmental water: An experimental and theoretical study.* Journal of Molecular Liquids, 2025: p. 127630.
3. Ezzati, R., *Derivation of pseudo-first-order, pseudo-second-order and modified pseudo-first-order rate equations from Langmuir and Freundlich isotherms for adsorption.* Chemical Engineering Journal, 2020. **392**: p. 123705.
4. Yaffar, D., J. Brenner, A.P. Walker, M.E. Craig, E. Vaughan, E. Marín-Spiotta, M. Matos, S. Rios, and M.A. Mayes, *The Freundlich isotherm equation best represents phosphate sorption across soil orders and land use types in tropical soils of Puerto Rico.* Biogeochemistry, 2025. **168**(2): p. 1-15.
5. Ángel, V.-O., T.-T. Candelaria, and G.-D. Ángel, *Parametric evaluation of a packed adsorption column to remove Cr (VI) using Langmuir and Freundlich with (LDF) and (QDF).* Desalination and Water Treatment, 2025: p. 101023.
6. Yang, H., S.J. Plathottam, K.R. Schell, T. Levin, and Z. Zhou, *Data-driven electricity price calibration based on Bayesian inference.* Energy Systems, 2025: p. 1-21.
7. Harbecke, J., J. Grunau, and P. Samanek, *Are the Bayesian Information Criterion (BIC) and the Akaike Information Criterion (AIC) Applicable in Determining the Optimal Fit and Simplicity of Mechanistic Models?* International Studies in the Philosophy of Science, 2024. **37**(1-2): p. 17-36.

8. Sun, Y., B.-Y. Peng, X. Wang, Y. Li, Y. Wang, Y. Zhang, S. Xia, and J. Zhao, *Adsorption and desorption mechanisms of oxytetracycline on poly (butylene adipate-co-terephthalate) microplastics after degradation: the effects of biofilms, Cu (II), water pH, and dissolved organic matter.* Science of the Total Environment, 2023. **863**: p. 160866.
